# SUMOylation of Matrix Protein M1 and Filamentous Morphology Collectively Contribute to the Replication and Virulence of Highly Pathogenic H5N1 Avian Influenza Viruses in Mammals

**DOI:** 10.1128/jvi.01630-21

**Published:** 2022-02-23

**Authors:** Jing Guo, Jianing Chen, Yuanyuan Li, Yanbing Li, Guohua Deng, Jianzhong Shi, Liling Liu, Hualan Chen, Xuyong Li

**Affiliations:** a College of Agronomy, Liaocheng University, Liaocheng, China; b State Key Laboratory of Veterinary Biotechnology, Harbin Veterinary Research Institute, Chinese Academy of Agricultural Sciences, Harbin, China; The Peter Doherty Institute for Infection and Immunity

**Keywords:** avian influenza virus, H5N1, M1, morphology, pathogenicity, SUMOylation

## Abstract

The matrix protein (M1) of influenza A virus plays an important role in replication, assembly, and budding. A previous study found that aspartic acid (D) at position 30 and alanine (A) at position 215 of M1 contribute to the high pathogenicity of H5N1 viruses in mice, and double mutations of D to asparagine (N) at position 30 (D30N) and A to threonine (T) at position 215 (A215T) in M1 dramatically attenuate H5N1 viruses in mice. However, the underlying mechanisms by which these M1 mutations attenuate the virulence of H5N1 viruses are unknown. Here, we found that the amino acid mutation A215T eliminates the SUMOylation of M1 by reducing its interaction with the host SUMO1 protein, significantly reducing the stability of M1, slowing the export of the M1-vRNP complex from the nucleus to the cytoplasm, and reducing viral replication in MDCK cells. We further found that the D30N mutation in M1 alters the shape of progeny viruses from filamentous to spherical virions. Our findings reveal an essential role for M1 215A SUMOylation and M1 30D-related filamentous morphology in the pathogenesis of avian influenza viruses, which could be targeted in novel antiviral drug designs.

**IMPORTANCE** Identification of the pathogenic mechanism of highly pathogenic avian influenza viruses in mammals is helpful to develop novel anti-influenza virus strategies. Two amino acid mutations (D30N and A215T) in M1 were found to collectively attenuate H5N1 influenza viruses in mice, but the underlying mechanism remained unknown. This study found that the A215T mutation significantly decreases the SUMOylation of M1, which in turn attenuates the replication of H5N1 virus in mammalian cells. The D30N mutation in M1 was found to change the virion shape from filamentous to spherical. These findings are important for understanding the molecular mechanism of virulence of highly pathogenic avian influenza viruses in mammals.

## INTRODUCTION

Influenza A virus is classified into different subtypes based on the antigenicity of the surface proteins hemagglutinin (HA) and neuraminidase (NA). All subtypes have been identified in avian species except for the H17N10 and H18N11 subtypes, which were found in bats (1). Avian influenza viruses, especially H5Nx viruses, are zoonotic agents recognized as public health threats (2). In 1996, a highly pathogenic H5N1 avian influenza virus was first detected in geese in China (3). A year later, a reassorted H5N1 virus caused disease outbreaks in poultry in Hong Kong (4) and the deaths of 6 of 18 infected persons, providing evidence that avian influenza viruses can cross the avian-human species barrier (5, 6). Human cases of H5N1 infection have frequently been reported worldwide in recent years and have resulted in more than 52.8% mortality (7). The H7N9 influenza virus that emerged in China in 2013 mutated to a highly pathogenic virus in chickens and posed an increasing threat to human public health (8–12). Moreover, the widespread H9N2 viruses that circulate in poultry have undergone mammalian adaptation and acquired increased transmissibility in ferrets (13, 14). In addition, the cocirculation of different subtypes in migratory waterfowl and domestic birds promotes the emergence of novel influenza reassortants, which threaten the poultry industry and human health (8, 15, 16). Therefore, revealing how these viruses influence virulence in humans and mammals will improve our understanding of the relationship between viruses and their hosts.

Several key amino acid substitutions have been identified as important molecular determinants of influenza virus replication, pathogenesis, and transmission in mammals. The E627K and D701N mutations in PB2 are known to increase the virulence and transmission of avian influenza virus in mammals (13, 17–20), whereas the Q226L and G228S mutations in HA contribute to enhanced transmission of avian influenza virus in mammals (21, 22). Glycosylation of HA also contributes to the virulence and antigenic properties of H5N1 viruses (23, 24). The roles of these key amino acids are usually closely related to certain host proteins. For example, when the ANP32a gene was knocked down in mice, the H7N9 virus lost the ability to acquire the E627K mutation in PB2 during the replication process and adopted another way of adapting to the host, which was to acquire the D701N mutation in PB2 (25, 26). The 627K in PB2 increases the polymerase activity, which is regulated by importin-α1 and importin-α7 (27).

SUMOylation is an important regulatory posttranslational protein modification mechanism in eukaryotic cells. The SUMOylation pathway is a multistep process similar to ubiquitination. It is initiated by the E1 SUMO-activating enzyme (SAE1 and SAE2), followed by the single E2 SUMO-conjugating enzyme (ubiquitin carrier 9 [UBC9]), and finally, but not necessarily, SUMO is covalently linked to target proteins by E3 SUMO ligase (28). SUMOylation can proceed in the presence of Ubc9 alone (29, 30). Ubc9 knockdown by RNA interference (RNAi) prevents target protein SUMOylation (31).

SUMOylation can regulate viral replication and protein functions. Several recent studies have indicated that the influenza A virus interacts extensively with host SUMOylation modification systems. Li et al. reported that a conserved lysine residue at position 612 of the PB1 protein is a SUMOylation site and is essential for the pathogenesis and transmission of influenza viruses (32). SUMOylation promotes the rapid growth of the influenza A virus by enhancing the stability of NS1 (33). SUMOylation of NP is essential for intracellular trafficking, resulting in accelerated virus replication (34). AIMP2 recruitment switches the modification of M1 from ubiquitination to SUMOylation, which promotes nuclear export of the M1-mediated viral ribonucleoprotein complex to increase viral replication (35).

In a previous study, Fan et al. investigated two H5N1 avian influenza viruses, A/duck/Guangxi/53/2002 (DKGX/53) and A/duck/Fujian/01/2002 (DKFJ/01), that have very similar genomes but exhibit different pathogenicity in mice: the 50% mouse lethal dose (MLD_50_) of DKGX/53 was 6.4 log_10_ 50% egg infectious dose (EID_50_), whereas that of DKFJ/01 was 0.9 log_10_ EID_50_ (36). By using reverse genetics, they found that the double mutations D30N and A215T in the M1 protein markedly attenuate DKFJ/01 virulence in mice (MLD_50_ of 0.9 log_10_ EID_50_ versus 4.8 log_10_ EID_50_). Moreover, they found that these two mutations in M1 collectively attenuated several other H5N1 viruses in mice (36). However, the underlying mechanism by which this viral amino acid combination influences the pathogenicity of the virus in mammals is currently unknown. In this study, we explored the mechanism underlying the role of M1 in H5N1 influenza virus pathogenicity in mammals.

## RESULTS

### Replication of H5N1 influenza viruses *in vitro*.

**FIG 1:**
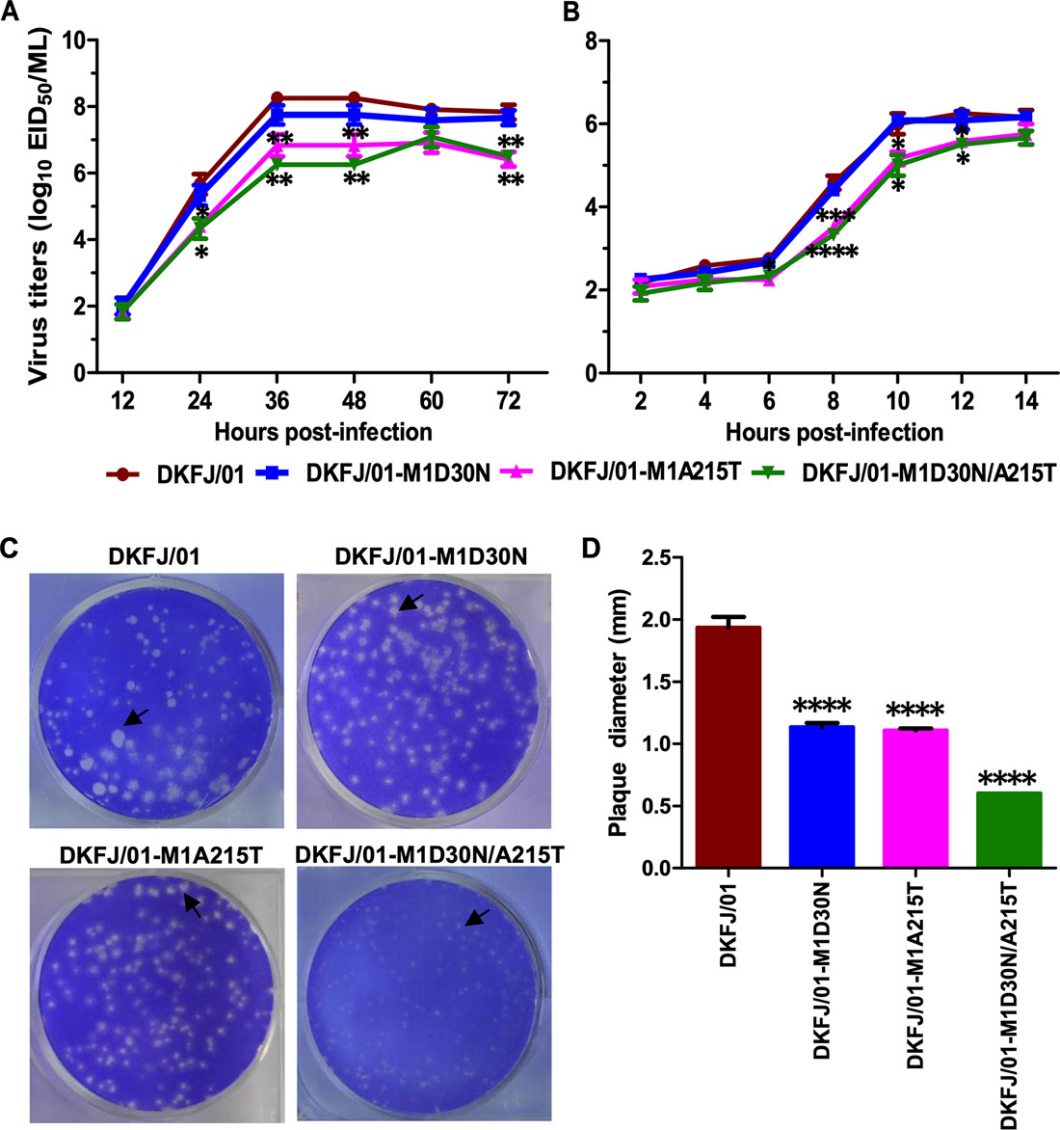
Replication of H5N1 influenza viruses in MDCK cells. (A) Multicycle replication curves of H5N1 viruses in cells. Monolayers of MDCK cells were inoculated at an MOI of 0.01 with viruses, and the culture supernatants were collected at the indicated time points and titrated in eggs. (B) One-cycle replication curves of H5N1 viruses in cells. Monolayers of MDCK cells were inoculated with viruses at an MOI of 1. The data shown are representative of three independent experiments (A and B). (C and D) Plaque formation assays. (C) Plaque assays were performed in MDCK cells under standard conditions with crystal violet staining. (D) Average plaque diameter for each virus. The diameters of 5 random plaques were measured for each virus. The significance was tested with a one-way ANOVA followed by a *t* test (A, B, and D). *, *P < *0.05; **, *P < *0.01; ***, *P < *0.001; ****, *P < *0.0001.

Fan et al. previously reported that two amino acid mutations (D30N and A215T) in M1 collectively attenuate H5N1 influenza viruses in mice (36). Generally, the virulence of a virus is positively related to the replication of that virus *in vitro* (32). Therefore, we further compared the growth kinetics of the wild-type virus (DKFJ/01) and its three mutants (DKFJ/01-M1D30N, DKFJ/01-M1A5T, DKFJ/01-M1D30N/A215T) in MDCK cells (Fig. 1A and B). Although all of the viruses replicated efficiently in MDCK cells, the viral titers of DKFJ/01-M1A215T and DKFJ/01-M1D30N/A215T were significantly lower than those of DKFJ/01 and DKFJ/01-M1D30N at 36 and 48 h postinfection (hpi) (Fig. 1A). In a one-cycle replication curve analysis, the viral titers of DKFJ/01-M1A215T and DKFJ/01-M1D30N/A215T in MDCK cells were significantly lower than those of DKFJ/01 and DKFJ/01-M1D30N at 8, 10, and 12 hpi (Fig. 1B). These data demonstrate that the amino acid change A215T in M1 has a more significant reducing effect on viral replication in MDCK cells than D30N.

To confirm the replication results, we performed a plaque assay and compared the plaque sizes of DKFJ/01 and the three mutants in MDCK cells. We found that the biggest plaque sizes of DKFJ/01, DKFJ/01-M1D30N, DKFJ/01-M1A215T, and DKFJ/01-M1D30N/A215T were 2.3 mm, 1.3 mm, 1.1 mm, and 0.6 mm, respectively (Fig. 1C), and their mean sizes were 1.9 mm, 1.2 mm, 1.1 mm, and 0.6 mm, respectively (Fig. 1D). These results indicate that, although both the D30N and A215T mutation in M1 play a role in attenuating viral replication, the A215T mutation has a more significant reducing effect than D30N on viral replication in MDCK cells.

### The M1 A215T mutation slows the nuclear export of vRNP complexes.

**FIG 2:**
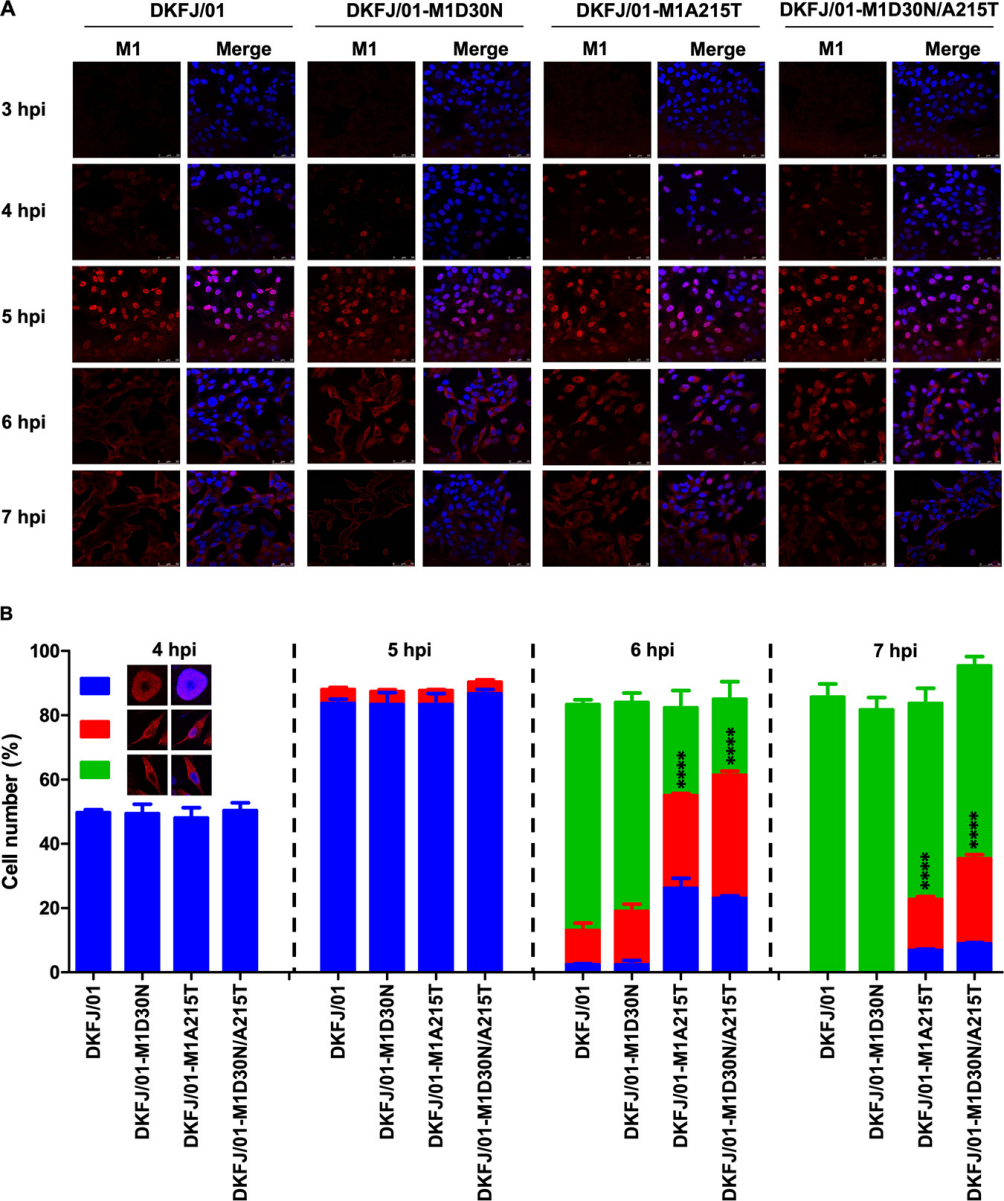
Cellular localization of the M1 protein in virus-infected cells at different time points. (A) MDCK cells were infected with the indicated viruses at an MOI of 5, and the localization of M1 was determined by observing the immunofluorescence at the indicated time postinfection. Cell nuclei were stained with DAPI. (B) M1 located in cells was quantified by counting cells (*n* = 100) infected with viruses under a microscope. Based on the confocal microscopy in panel A, the localization of M1 was categorized into three types: clear nuclear localization (blue), simultaneous localization in the cytoplasm and nucleus (red), and predominantly cytoplasmic localization (green). The data shown are means and SD. The numbers of cells with target proteins observed in the nuclei (including in both the cytoplasm and the nucleus) of the mutant virus-infected samples and the DKFJ/01-infected samples were compared. Statistical analysis was performed by using a one-way ANOVA with GraphPad Prism 8 software. ****, *P < *0.0001.

The replication of influenza viruses in cells involves several steps, and the M1 protein is involved in the transport and assembly of viral ribonucleoprotein (vRNP) and the budding of progeny virions (37). The newly synthesized M1 protein is transported from the cytoplasm to the nucleus, where it forms the M1-vRNP complex, facilitating vRNP export from the nucleus and subsequent virion assembly and virus budding (38–40). We therefore investigated whether the mutations in M1 affect these processes. The fluorescence signal of M1 was not detectable at 3 hpi and was detected in the nucleus rather than in the cytoplasm at 4 hpi, indicating that newly synthesized M1 protein was imported into the nucleus from the cytoplasm (Fig. 2A). The number of cells with M1 accumulation in the nucleus gradually increased and peaked at 5 hpi, but no clear difference was observed in the cells infected with the four different viruses (Fig. 2B), indicating that the mutations in M1 did not affect its nuclear import. At 6 hpi, the M1 protein was transported from the nucleus to the cytoplasm in about 70% of the DKFJ/01-infected cells and 65% of the DKFJ/01-M1D30N-infected cells, but this M1 nuclear export was only observed in 27% of the DKFJ/01-M1A215T-infected cells and in 24% of the DKFJ/01-M1D30N/A215T virus-infected cells (Fig. 2A and B). At 7 hpi, M1 was transported to the cytoplasm from the nucleus in nearly all of the DKFJ/01- and DKFJ/01-M1D30N-infected cells but was still observed in the nuclei of 23% of the DKFJ/01-M1A215T-infected cells and 35% of the DKFJ/01-M1D30N/A215T-infected cells (Fig. 2A and B). In addition, we found that the M1 of DKFJ/01 and DKFJ/01-M1D30N but not that of DKFJ/01-M1A215T and DKFJ/01-M1D30N/A215T showed clear localization at the plasma membrane (Fig. 2A). These findings indicate that the D30N and A215T mutations have no obvious effect on the nuclear import of M1, but the mutation A215T dramatically slows the nuclear export of M1 in virus-infected cells.

The vRNP complex comprises eight genomic vRNAs, each bound to nucleoprotein and polymerase complexes. The transportation of the polymerase protein between the cytoplasm and nucleus affects the efficiency of influenza virus replication (41, 42).

**FIG 3:**
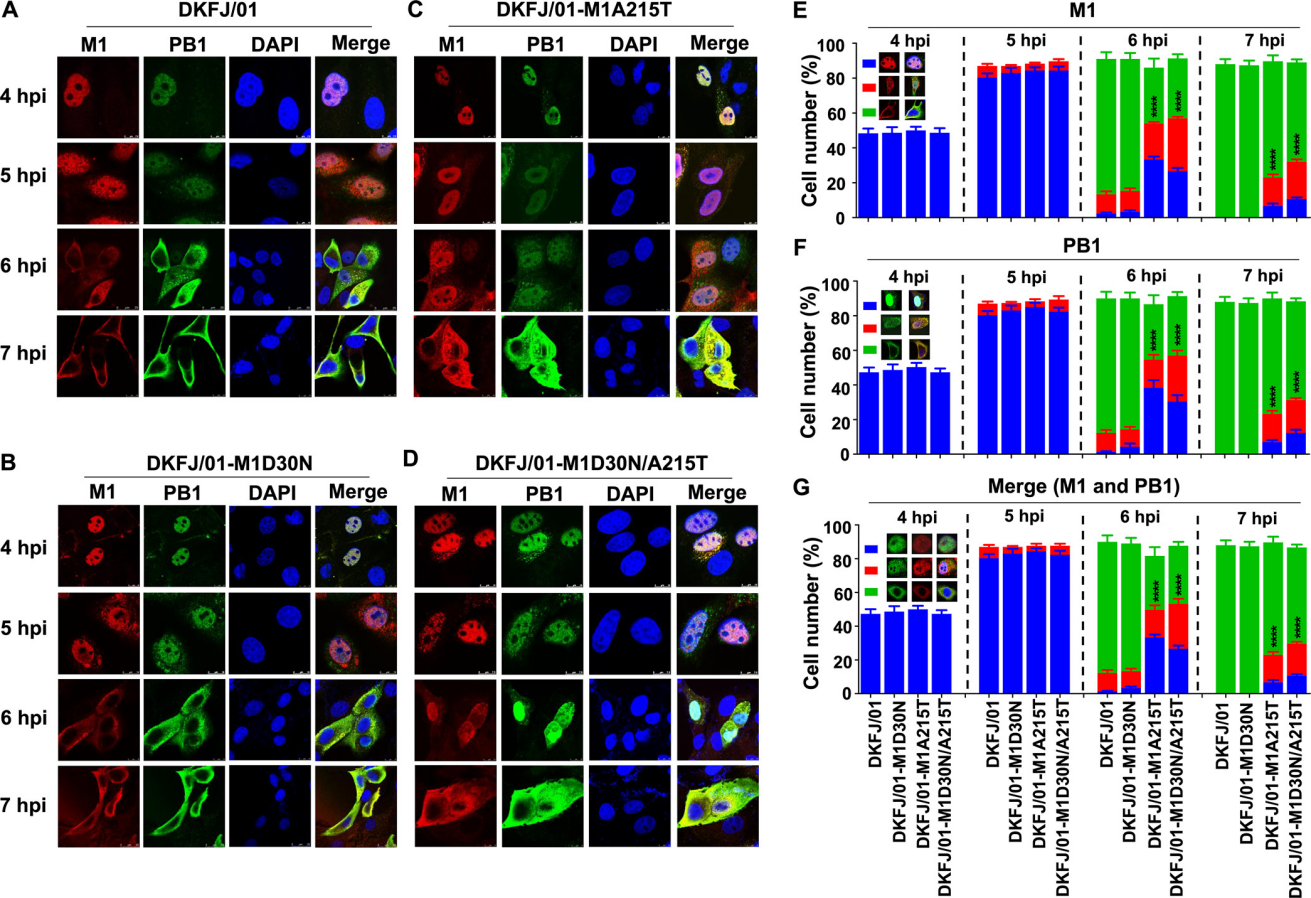
Cellular colocalization of M1 and PB1 in virus-infected cells at different time points. MDCK cells were infected with DKFJ/01 (A), DKFJ/01-M1D30N (B), DKFJ/01-M1A215T (C), or DKFJ/01-M1D30N/A215T (D) at an MOI of 5, and the colocalization of M1 and PB1 was determined by use of confocal microscopy at the indicated time points. Cell nuclei were stained with DAPI. PB1 located in cells was quantified by counting cells (*n* = 100) infected with viruses under a microscope (F). Based on the confocal microscopy in panel A, the localization of M1 (E), PB1 (F), or M1+PB1 (G) was categorized into three types: clear nuclear localization (blue), simultaneous localization in the cytoplasm and nucleus (red), and predominantly cytoplasmic localization (green). The number of cells with target proteins observed in the nuclei (including in both the cytoplasm and the nucleus) of the mutant virus-infected samples and the DKFJ/01-infected samples was compared. The values shown are means and SD of the results of three independent experiments. Statistical analysis was performed using a one-way ANOVA with GraphPad Prism 8 software. ****, *P < *0.0001.

To investigate whether the mutations in M1 affect the nuclear import and export of the vRNP complex, we examined the cellular colocalization of M1 and PB1 in cells infected with DKFJ/01 and its three mutants at different time points. Similar to M1 (Fig. 3A to G), in nearly half of the virus-infected cells, PB1 had accumulated in the nucleus at 4 hpi, and by 5 hpi, more than 85% of the infected cells showed nuclear accumulation of PB1 (Fig. 3A to D and F), indicating that the nuclear import of vRNP was not affected by the mutations in M1. In contrast, a clear difference in PB1 nuclear export was observed in the cells infected with the four viruses. At 6 hpi, the translocation of PB1 from the nucleus to the cytoplasm was observed in 78% of the DKFJ/01-infected cells and 76% of the DKFJ/01-M1D30N-infected cells, but in only 32% of the DKFJ/01-M1A215T-infected cells and 34% of the DKFJ/01-M1D30N/A215T-infected cells (Fig. 3A to D and F). At 7 hpi, the PB1 in all of the DKFJ/01- and DKFJ/01-M1D30N-infected cells had been exported to the cytoplasm, but the PB1 remained in the nucleus of 23% and 31% of the DKFJ/01-M1A215T- and DKFJ/01-M1D30N/A215T-infected cells, respectively (Fig. 3A to D and F). The changes in the numbers of cells in which M1 and PB1 were colocalized at different time points showed a pattern similar to that of M1 and PB1 localization (Fig. 3A to D and G). These results indicate that the amino acid mutation A215T in M1 influences the nuclear export of the vRNP complex.

To confirm these results, the vRNA level (NP gene) in the nucleus, cytoplasm, and whole cell of the virus-infected samples was measured by using quantitative reverse transcription-PCR (qRT-PCR), and the values were compared with those for the DKFJ/01-infected cells. At 3, 4, and 5 hpi, the levels of vRNA in different parts of the cells were comparable among the samples infected with the four different viruses (see Fig. S1A to C in the supplemental material). However, at 6 hpi and 7 hpi, the vRNA levels in the nucleus of the DKFJ/01-M1A215T- and DKFJ/01-M1D30N/A215T-infected cells were significantly higher than that in the DKFJ/01-infected cells (Fig. S1D and E). These results further confirm that the amino acid change of A215T in the M1 protein slows the nuclear export of vRNP and explain the significant growth difference between DKFJ/01 and viruses carrying the A215T mutation in M1.

To investigate whether the mutations in M1 affect the polymerase activity, we performed a dual-luciferase reporter assay to compare the activity levels of the viral RNP complex. The data showed that the amino acid mutations at positions 30 and 215 in M1 do not affect viral polymerase activity (Fig. S2).

Taken together, our results suggest that the A215T mutation in the M1 protein mainly attenuates the replication of H5N1 virus in mammalian cells by slowing the nuclear export of vRNP in the virus-infected cells.

### The A215T mutation affects the SUMOylation of the M1 protein.

**FIG 4:**
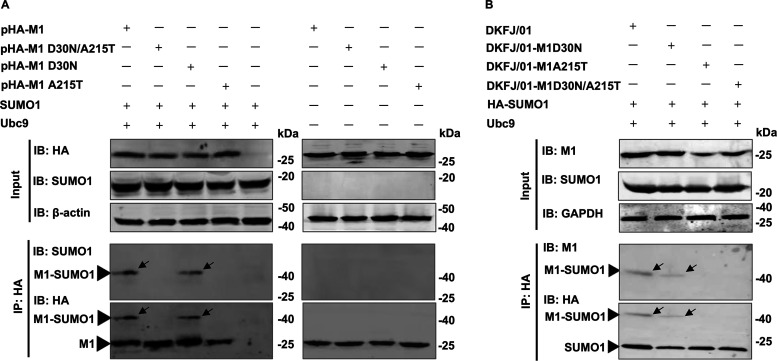
The mutation A215T in M1 affects the modification of the M1 protein by SUMO1. (A) Plasmids expressing HA-tagged wild-type or mutant M1 of H5N1 influenza virus were cotransfected with Ubc9- and SUMO1-expressing plasmids into 293T cells. Forty-eight hours posttransfection, the lysates were immunoprecipitated with anti-HA agarose, followed by Western blotting analysis using antibodies against HA and SUMO1. (B) 293T cells were transfected with plasmids encoding HA-SUMO1 and Ubc9. At 24 h posttransfection, the cells were infected with DKFJ/01, DKFJ/01-M1D30N, DKFJ/01-M1A215T, or DKFJ/01-M1D30N/A215T at an MOI of 1. Eighteen hours later, the cell lysates were immunoprecipitated with anti-HA agarose, followed by Western blotting analysis using antibodies against M1 and the HA tag.

SUMOylation, a posttranslational modification, plays a specific role in the replication and virulence of influenza virus and other pathogens (32, 43, 44). Given the importance to virus replication and virulence of the SUMO1 protein in the posttranslational modification of influenza virus proteins, we investigated whether the M1 protein of DKFJ/01 virus and its mutants could interact with the SUMO1 protein and be SUMOylated. 293T cells were cotransfected with hemagglutinin (HA)-tagged M1 expression plasmids (pHA-M1, pHA-M1D30N, pHA-M1A215T, or pHA-M1D30N/A215T), along with SUMO1 and Ubc9 (a unique and essential gene in the E2 SUMO conjugation system) expression plasmids (28). Forty-eight hours posttransfection, the cells were lysed and immunoprecipitated with anti-HA agarose and were then subjected to Western blot analysis. A clear SUMOylated M1 band (M1-SUMO1) (approximately 43 kDa) that has a higher molecular weight than the M1 band (27 kDa) was observed in the pHA-M1- and pHA-M1D30N-transfected samples but was not observed in the pHA-M1A215T- or pHA-M1D30N/A215-transfected samples (Fig. 4A; Fig. S4). To confirm these results, 293T cells transiently overexpressing HA-SUMO1 and Ubc9 were infected with DKFJ/01, DKFJ/01-M1D30N, DKFJ/01-M1A215T, or DKFJ/01-M1D30N/A215T virus at a multiplicity of infection (MOI) of 1, and 10 h later, cell lysates were immunoprecipitated with anti-HA agarose. A band of M1-SUMO1 was detected in DKFJ/01 and DKFJ/01-M1D30N virus-infected cells, but not in cells infected with DKFJ/01-M1A215T or DKFJ/01-M1D30N/A215T (Fig. 4B). These results indicate that the amino acid mutation A215T abolishes the SUMOylation of M1 protein.

**FIG 5:**
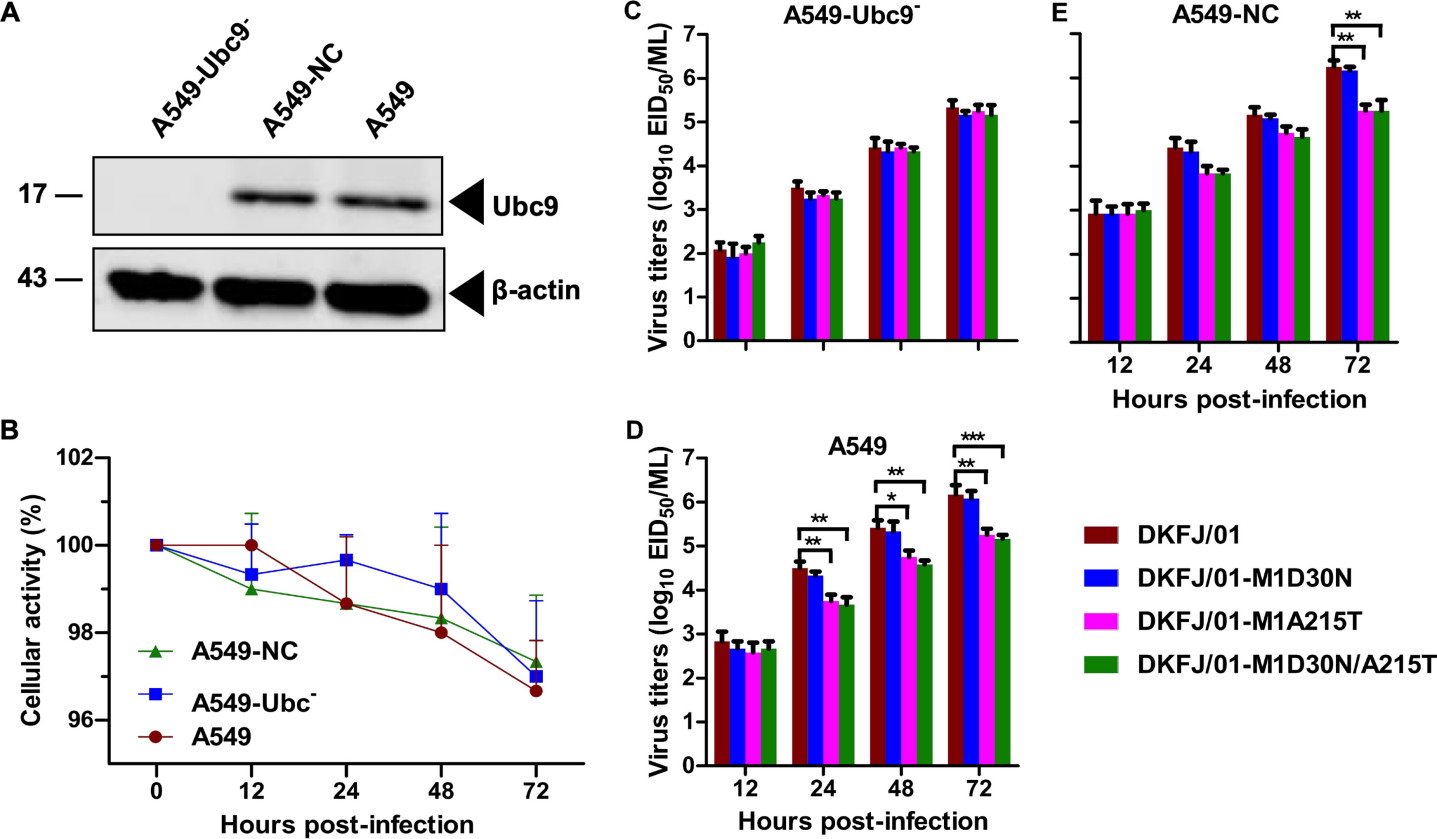
Silencing of the SUMOylation system affects virus replication. (A) Western blot analysis of A549-Ubc9^−^ cells. Cell lysates were prepared from siUbc9 cells (A549-Ubc9^−^), control siRNA cells (A549-NC), and naive A549 cells and subjected to Western blot analysis. (B) Knocking down Ubc9 did not affect cell viability. (C to E) Comparison of virus production rates. The three cell lines mentioned above were infected with the influenza A virus at an MOI of 0.01. Culture supernatants were collected at the indicated time points and then titrated in eggs. Data shown are means and SD for three independent experiments; significance was assessed with a one-way ANOVA followed by a *t* test. *, *P < *0.05; **, *P < *0.01; ***, *P < *0.001.

To investigate whether SUMOylation is involved in the life cycle of the influenza virus, an RNAi approach was employed to suppress the SUMOylation system in the cells by silencing Ubc9 (31, 44, 45). The expression of Ubc9 protein was nearly undetectable in the siUbc9 construct-transfected cells, and Ubc9 gene knockdown did not affect cell activity (Fig. 5A and B). Ubc9 knockdown cells (A549-Ubc9^−^), control small interfering RNA (siRNA) cells (A549-NC), and untransfected A549 cells (naive A549) were infected with DKFJ/01, DKFJ/01-M1D30N, DKFJ/01-M1A215T, or DKFJ/01-M1D30N/A215T at an MOI of 0.01. The replication titers of the DKFJ/01 and DKFJ/01-M1D30N viruses were significantly higher than those of the DKFJ/01-M1A215T and DKFJ/01-M1D30N/A215T viruses in the A549 and A549-NC cells at certain time points, but the replication titers of all four viruses were the same or showed little difference in the A549-Ubc9^−^ cells (Fig. 5C to E). These results demonstrate that the mutation A215T prevents the M1 protein from being SUMOylated and thereby attenuates the replication of the H5N1 influenza virus in mammalian cells.

### The A215T mutation reduces the stability of the M1 protein.

**FIG 6:**
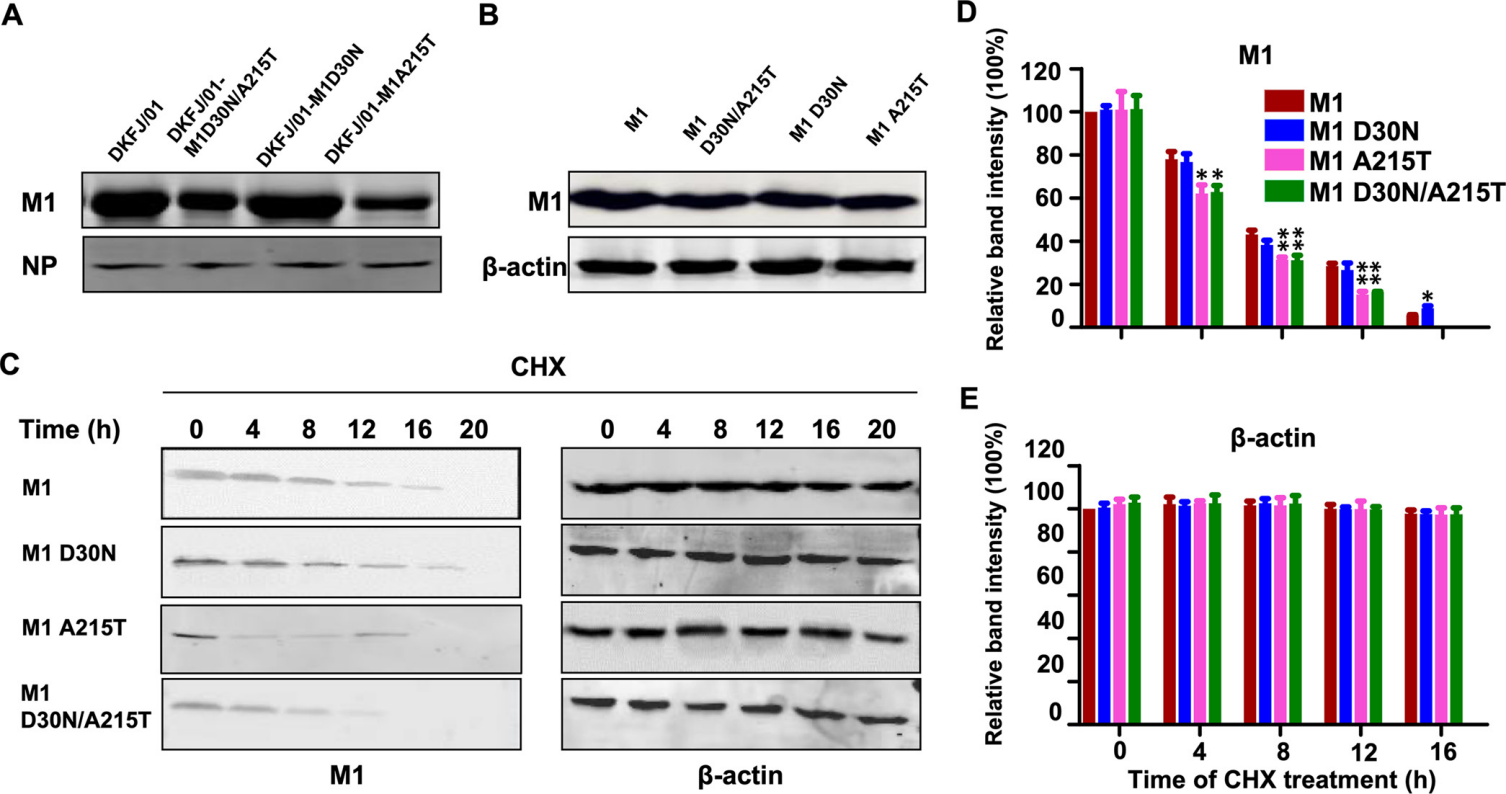
The mutation A215T in M1 decreases the stability of the M1 protein. (A) MDCK cells were infected with the wild-type and mutant viruses at an MOI of 1, and the level of viral proteins was analyzed by Western blotting 10 h postinfection. (B) 293T cells were transfected with wild-type or mutant M1. The cells were harvested, and lysates were subjected to Western blotting with an anti-M1 antibody. (C) 293T cells were transfected with wild-type or mutant M1 and then treated with 10 nM CHX. The cells were harvested at various times, and lysates were subjected to Western blotting with an anti-M1 antibody. (D and E) Quantification of Western blotted M1 or β-actin in panel C. The band intensities of the Western blots from three assays were quantified by using ImageJ software and compared with the value of the DKFJ/01-M1-transfected sample. Statistical analysis was performed by using a one-way ANOVA with GraphPad Prism 8 software. *, *P < *0.05; **, *P < *0.01; ***, *P < *0.001.

SUMOylation is important for protein stability (33). Our results indicate that the mutation A215T abolishes the SUMOylation of the M1 protein. We therefore investigated whether the M1 protein of DKFJ/01-M1A215T and DKFJ/01-M1D30N/A215T is less stable than that of DKFJ/01 and DKFJ/01-M1D30N. MDCK cells were infected with the test viruses at an MOI of 1, and the levels of the SUMO1, M1, and NP proteins in the virus-infected cells were measured at 10 hpi. We found that the SUMO1 and NP levels were comparable among the cells infected with the four viruses (Fig. 6A; Fig. S3), but that the M1 protein level in the DKFJ/01-M1A215T- and DKFJ/01-M1D30N/A215T-infected cells was clearly less intense than that in the DKFJ/01- and DKFJ/01-M1D30N-infected cells (Fig. 6A). The M1 expression levels were comparable in cells transfected with different plasmids carrying wild-type M1 or the M1 mutants (Fig. 6B), indicating that the mutations D30N and A215T did not affect the expression of M1. To investigate the stability of the M1 protein in cells transfected with different plasmids, 293T cells were treated with the protein translation inhibitor cycloheximide (CHX) and were then harvested at 0, 4, 8, 12, 16, and 20 h after treatment to check the M1 protein levels by Western blotting analysis. We found that the protein levels of M1 in all of the cells transfected with the four plasmids declined over time and became undetectable at 20 h post-CHX treatment, but the M1 protein levels in the cells transfected with pM1A215T and pM1D30N/A215T declined significantly faster than those in the cells transfected with pM1 and pM1D30N (Fig. 6C and D), indicating that the amino acid mutation A215T decreases the stability of the M1 protein of influenza virus.

**FIG 7:**
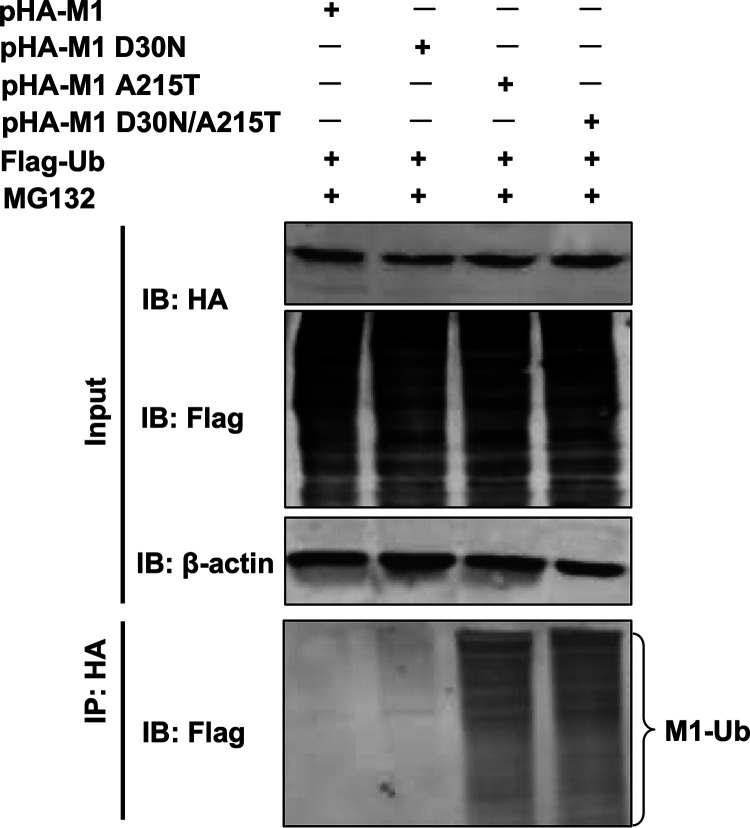
Ubiquitin degradation of the M1 protein. 293T cells were cotransfected with HA-tagged wild-type or mutant M1 and Flag-Ub-expressing plasmids and treated with MG132 (a 26S proteasome inhibitor). Then, the lysates were immunoprecipitated with anti-HA agarose, followed by Western blotting analysis using antibodies against Flag.

SUMO1 and ubiquitin compete for binding to the target protein. When the SUMO1 protein does not sumoylate the target protein, ubiquitin has the opportunity to bind and thereby degrade the target protein (31, 46). To investigate whether the degradation of M1 protein bearing the mutation A215T is linked to ubiquitination, 293T cells were cotransfected with HA-tagged wild-type or mutant M1 plasmids and a Flag-tagged ubiquitin-expressing plasmid, and the cells were then treated with MG132 (a 26S proteasome inhibitor). The lysates were immunoprecipitated with anti-HA agarose, followed by Western blotting. We found that the ubiquitination levels of M1 proteins bearing the A215T single mutation and the D30N/A215T double mutations were significantly higher than that of wild-type M1 protein or M1 proteins bearing the D30N single mutation (Fig. 7). These data indicate that the amino acid mutation A215T prevents SUMOylation of M1 and thereby promotes the interaction between M1 and ubiquitin, facilitating M1 degradation.

In summary, the A215T mutation but not the D30N mutation in M1 prevents SUMOylation and promotes ubiquitination to degrade the M1 protein, which leads to slow export of the M1-vRNP complex from the nucleus to the cytoplasm and significantly attenuates the replication of H5N1 viruses in mammalian cells.

### The D30N mutation in M1 affects viral morphology.

**FIG 8:**
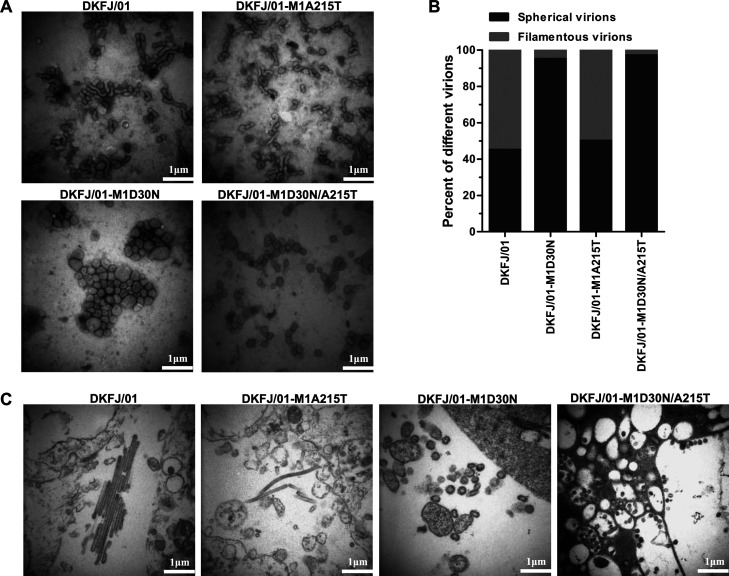
Virus morphology observed by electron microscopy. (A) Electron micrographs were obtained to observe viral morphogenesis. The viruses grown in eggs were concentrated, negatively stained, and examined under an electron microscope. (B) Statistical analysis of virus morphology. Twenty fields of each virus were observed under a scanning electron microscope, and the filamentous and spherical virions were counted. (C) Ultrastructural analysis of virus-infected cells. MDCK cells infected with the indicated viruses were examined under a transmission electron microscope at 10 hpi.

Some studies have reported that the M1 protein plays an important role in determining the morphology of influenza viruses, and morphology can affect viral transmission and virulence (47–50). Therefore, we investigated whether the D30N and A215T mutations affect the morphology of the DKFJ/01 virus. The viruses were cultured in eggs and purified by sucrose density gradient centrifugation. The purified viruses were then observed under an electron microscope. The DKFJ/01 sample comprised 55% filamentous virions and 45% spherical virions, and the DKFJ/01-M1A215T sample comprised 50% filamentous and 50% spherical virions. In contrast, the DKFJ/01-M1D30N sample comprised 5% filamentous virions and 95% spherical virions, and the DKFJ/01-M1D30N/A215T sample comprised 3% filamentous and 97% spherical virions (Fig. 8A and B). To confirm this result, MDCK cells were infected with the DKFJ/01 virus and its three mutants, and ultrathin sections of the cells and progeny viruses were prepared at 10 hpi for observation by using transmission electron microscopy. Filamentous progeny virions were observed in the DKFJ/01- and DKFJ/01-M1A215T virus-infected samples, whereas spherical virions were mainly found in the DKFJ/01-M1D30N- and DKFJ/01-M1D30N/A215T virus-infected samples (Fig. 8C). These findings indicate that the mutation D30N, but not A215T, in M1 significantly affects viral morphology.

## DISCUSSION

H5N1 influenza viruses have circulated worldwide, acquired complicated biological properties, and attracted extensive attention due to their lethal impact on mammals, including humans (3, 51). A previous report showed that the amino acids D30 and A215 in the M1 protein contribute to the virulence of H5N1 avian influenza viruses in mice and the substitutions D30N and A215T dramatically attenuate the virulence (36). Here, we found that the mutation A215T in M1 reduces viral titers in mammalian cells, as the M1 A215T mutant slowed the M1-vRNP complex export from the nucleus, thus impairing the viral life cycle. We further revealed that amino acid 215 in M1 affects the interaction of the M1 protein with SUMO1. SUMO conjugase Ubc9 transfers SUMO1 to the target protein, and SUMOylation can proceed in the presence of Ubc9 alone. Ubc9 knockdown by RNAi prevents target protein SUMOylation (31). When we knocked down the Ubc9 gene in cells, the M1 protein was not modified by SUMO1, and the replication titers of the wild-type virus and mutant viruses in cells decreased similarly. We also found that the A215T mutation accelerates the degradation of M1 through ubiquitin modification. Moreover, we found that the mutation D30N in the M1 protein dramatically increases the ratio of spherical particles.

Host proteins are involved in the regulation of influenza virus replication and virulence. SUMO1 is an important posttranslational modification protein and plays a key role in regulating protein activity, localization, and stability of viral proteins. The PB1, NP, M1, and NS1 proteins have been shown to be SUMOylated in transfected and influenza virus-infected cells (32, 34, 44, 52). For different target proteins, the recognition motif of SUMOylation is different. Generally, the lysine of the target protein is the recognition site of SUMO1 (53). However, other amino acids also affect target protein modification by SUMOylation. Simon et al. reported that SUMO1 modification of the lamin A tail was reduced by two familial partial lipodystrophy (FPLD)-causing mutations, G465D and K486N, and by single mutations in the acidic residues E460 and D461 (54). Buschmann et al. reported that Ubc9 associates with Mdm2 only when amino acids 40 to 59 in the N terminus of Mdm2 are present (55). In the present study, we confirmed that 215A plays an important role in the SUMOylation of the M1 protein and that the A215T mutation prevents the SUMOylation of the M1 protein. These findings suggest that target proteins may have different SUMOylation recognition motifs, possibly because of their differences in amino acid sequence or conformation.

A previous study found that SUMOylated M1 interacts with vRNP to form the RanGTP-Crm1-NS2-M1-vRNP complex, which mediates the nuclear export of vRNP (38, 39). Wu et al. demonstrated that abolishing SUMOylation at the C domain of M1 reduces the formation of the M1-vRNP complex and thereby inhibits the nuclear export of vRNP (44). Here, we demonstrated that the amino acid mutation A215T in M1 prevents the SUMOylation of the M1 protein, thus slowing the export of M1-vRNP from the nucleus to the cytoplasm and reducing the replication efficiency of the virus. These results suggest that small molecules that block the SUMOylation of M1 could be designed as potential drug targets to inhibit influenza virus infection of mammalian cells.

The M1 protein plays a crucial role in the assembly and budding of influenza virus and is the main viral determinant of filamentous morphology (47, 56–58). Previous studies reported that swapping the M segment from the spherical A/Puerto Rico/8/1934 virus with the M segment of the filamentous A/Udorn/1972 virus enabled the conversion of a spherical particle into a filamentous particle (56). Kong et al. reported that the amino acid at position 156 of M1 is important for determining the morphology and transmissibility of H7N9 influenza viruses (47). Here, we found that the mutation D30N in M1 changes the virus from filamentous to spherical and, together with the A215T mutation, reduces the virulence of H5N1 virus. These findings indicate that amino acids at multiple sites in M1 could affect the morphology and biologic properties of influenza viruses.

In conclusion, our study showed that the A215T mutation significantly decreases the SUMOylation of the M1 protein, which in turn causes a striking reduction in progeny virus production and weakened replication of the H5N1 virus in mammalian cells. In addition, the D30N mutation was shown to mainly change progeny virus morphology from filamentous to spherical virions. These results revealed the mechanism of attenuation of H5N1 virus bearing the double mutation D30N and A215T in M1 in mice. Our findings provide new insights into the pathogenesis of H5N1 virus and the development of novel antiviral drugs to better control cross-species infection with avian influenza virus.

## MATERIALS AND METHODS

### Biosafety facility.

All experiments with live H5N1 viruses were conducted within the enhanced animal biosafety level 3 (ABSL3+) facility at the Harbin Veterinary Research Institute (HVRI) of the Chinese Academy of Agricultural Sciences (CAAS) approved for such use by the Ministry of Agriculture and Rural Affairs of the People's Republic of China.

### Cell lines and viruses.

Madin-Darby canine kidney (MDCK), human embryonic kidney (293T), and human lung adenocarcinoma epithelial (A549) cell lines were purchased from the Cell Resource Center of the Shanghai Institute of Life Sciences and were maintained in our laboratory. The MDCK and 293T cell lines and viruses were grown in Dulbecco's modified Eagle's medium (DMEM) containing 10% fetal bovine serum (FBS) and antibiotics. The A549 cells were grown in an F-12K nutrient mixture containing 10% FBS and antibiotics. All cells were cultured at 37°C with 5% CO_2_.

The viruses used in this study, DKFJ/01, DKFJ/01-M1D30N, DKFJ/01-M1A215T, and DKFJ/01-M1D30N/A215T, were generated by reverse genetics (36), propagated in specific pathogen-free (SPF) embryonated chicken eggs, and stored at −80°C until use.

### Virus growth kinetics.

In a multicycle replication curve experiment, monolayer cultures of MDCK cells were infected with DKFJ/01, DKFJ/01-M1D30N, DKFJ/01-M1A215T, or DKFJ/01-M1D30N/A215T virus at a multiplicity of infection (MOI) of 0.01. In the one-cycle replication curve experiment, monolayer cultures of MDCK cells were infected with the wild-type and mutant viruses at an MOI of 1. Virus-containing culture supernatants were collected at various times postinfection and titrated in eggs. The growth data shown are the average results of three independent experiments.

### Plaque assay.

Monolayers of MDCK cells in 6-well dishes were infected with the virus in Opti-MEM and incubated at 37°C for 1 h. The cells were then washed and overlaid with MEM containing 0.8% low-melting-point agarose. After 2 days, the cells were fixed with 70% ethanol and stained with 0.1% crystal violet. Plaque size was measured by using a fine-scale magnifying comparator (6×), as described previously (59). The probability of a significant difference in plaque size between viruses was computed using a one-way analysis of variance (ANOVA) followed by a *t* test.

### Fluorescence focus assay.

MDCK cells grown on glass-bottom dishes to 70% to 80% confluence were infected with DKFJ/01, DKFJ/01-M1D30N, DKFJ/01-M1A215T, or DKFJ/01-M1D30N/A215T virus at an MOI of 5. At the indicated time points, cells were fixed in phosphate-buffered saline (PBS) containing 4% paraformaldehyde for 30 min and permeabilized with PBS containing 0.5% Triton X-100 for 30 min. The cells were then blocked with 5% skimmed milk in PBS and incubated with mouse monoclonal antibody against M1 or rabbit monoclonal antibody against PB1 at room temperature for 45 min. The cells were then washed three times with PBS and incubated for 45 min with rhodamine-coupled donkey anti-mouse antibody for M1 detection or fluorescein isothiocyanate (FITC)-coupled goat anti-rabbit antibody for PB1 detection. After incubation with secondary antibodies, the cells were washed three times with PBS and incubated with 4′,6-diamidino-2-phenylindole (DAPI) for 10 min. The cells were observed with a laser scanning confocal microscope (Leica). If there was red or green fluorescence in the nucleus, the M1 or PB1 was in the nucleus; if there was no red or green fluorescence in the nucleus, the M1 or PB1 was not in the nucleus or had exited the nucleus. Protein nuclear localization was determined by counting the virus-infected cells (*n* = 100) with the test proteins. The results shown represent three independent experiments.

### Nuclear and cytosolic vRNA assessment by use of real-time qPCR.

MDCK cells were infected with DKFJ/01, DKFJ/01-M1D30N, DKFJ/01-M1A215T, or DKFJ/01-M1D30N/A215T virus at an MOI of 5. The cells were collected at the indicated time points, and the nuclear and cytosolic fractions were separated by using the BioVision kit according to the manufacturer's instructions. Cellular, nuclear, and cytosolic vRNA was measured by using qRT-PCR with NP-specific primers: F, 5′- CCCAAGGCACCAAACGA-3′, and R, 5′- TTCCACCAACCATTCTTCCA-3′. The qPCR primers for actin were 5′-GTCGTACCACTGGCATCGTG-3′ (F) and 5′- TCTCAGCTGTGGTGGTGAAG-3′ (R).

### Western blot and immunoprecipitation analyses.

vRNA was extracted from the stock viruses by using the QIAamp viral RNA minikit (Qiagen, Valencia, CA). cDNA was synthesized from vRNA by using a RevertAid first-strand cDNA synthesis kit (Fermentas) with gene-specific primers. M1 was amplified from virus cDNA by PCR with a 5′ primer that introduced an HA tag with a NheI site and a 3′ primer that introduced a CalI site. HA-M1, HA-M1 D30N, HA-M1 A215T, and HA-M1 D30N/A215T were cloned into the pCAGGS vector (60). The open reading frames (ORFs) of SUMO1 and Ubc9 were amplified from human A549 cDNA by PCR with a 5′ primer with a NheI site and a 3′ primer with a CalI site and were cloned into pCAGGS (28). SUMO1 was further subcloned into pCAGGS with an HA tag at the C terminus. The ORFs for ubiquitin (Ub) were amplified from human A549 cDNA by PCR with a 5′ primer that introduced a Flag tag with a NheI site and a 3′ primer that introduced a CalI site. Sequencing was performed to confirm the identity of all plasmids (31, 46). The 239T cells were (i) cotransfected with the expression plasmids of Ubc9, SUMO1, and HA-M1, (ii) cotransfected with the expression plasmids of Ubc9 and HA-SUMO1 and then infected with four viruses, and (iii) cotransfected with the expression plasmids of Flag-Ub and HA-M1 and treated with MG132 (M7449; Sigma) 18 h after transfection. Transfection was conducted using Lipofectamine LTX (Invitrogen), and each plasmid concentration was 1.5 μg/μL. Forty-eight hours posttransfection, the cells were lysed with lysis buffer containing 20 nM *N*-ethylmaleimide (NEM) and complete proteinase inhibitor (Roche) and then subjected to immunoprecipitation by using a ProFound HA tag immunoprecipitation (IP)/co-IP kit (Pierce), followed by Western blotting analysis.

Protein samples were mixed with 5 × sample buffer and loaded on SDS-PAGE gels, and then the proteins were transferred onto nitrocellulose membranes. The membranes were blocked for 1 h at room temperature with PBS containing 5% skim milk. The membranes were then incubated with primary antibodies for 1 h at room temperature. To detect M1, HA, Flag, or SUMO1, anti-M1 mouse polyclonal (1:500), anti-HA mouse monoclonal (1:1,000), anti-Flag rabbit monoclonal (1:1,000), and anti-SUMO1 rabbit monoclonal (1:500) antibodies were used. After three washes with PBS-Tween (PBS-T) for 5 min each time, the membranes were incubated with anti-mouse (1:5,000) secondary antibodies and anti-rabbit (1:5,000) secondary antibodies conjugated with horseradish peroxidase (Invitrogen) for 1 h at room temperature. After three washes with PBS-T for 5 min each time, the blots were developed using the Odyssey infrared imaging system (Li-Cor).

### siRNAs.

One 21-nucleotide siRNA against human Ubc9 (AAACAGAUCCUAUUAGGAATT) was synthesized commercially (Invitrogen), and a negative-control siRNA (SI03650318) was purchased from Qiagen (Shanghai, China). Transfection was conducted using Lipofectamine LTX (Invitrogen), and the siRNA concentration was 80 nM in A549 cells. Cell activity was determined using Cell Proliferation Kit I (catalog number 11465007001; Roche). At 24 h after transfection with siUbc9, A549 cells were infected with virus at an MOI of 0.01. Virus-containing culture supernatant was collected at various time points and titrated in eggs.

### Protein stability.

The M1 was amplified from viral RNA by RT-PCR with a 5′ primer that introduced a NheI site and a 3′ primer that introduced a CalI site. M1, M-D30N, M1-A215T, or M1-D30N/A215T were cloned into pCAGGS. The 239T cells were transfected with the four plasmids expressing these genes and were treated with the protein synthesis inhibitor CHX (Sigma) 8 h after transfection. Lipofectamine LTX was purchased from Invitrogen, and the concentration of the plasmids was 150 ng/μL. The cells were collected at 0, 4, 8, 12, 16, and 20 h after CHX was added and analyzed by Western blotting. The band intensities of the Western blots from three assays were quantified using ImageJ software, and the band intensity values of each gel were compared with the value of the DKFJ/01-M1-transfected sample.

### Luciferase assay of polymerase activity.

The protein expression plasmids for PB2, PB1, PA, NP, M1, and M1 mutants were generated by inserting their cDNA sequences into a pcDNA3.1 vector. Lipofectamine LTX was purchased from Invitrogen, and the concentration of the plasmids was 500 ng/μL. A luciferase activity assay was performed using a luciferase activity assay kit from Promega according to the manufacturer's protocol. Cell extracts were harvested 48 h posttransfection, and the luciferase activity was assayed by using the luciferase assay system. The assay was standardized against *Renilla* luciferase activity. All experiments were performed in triplicate.

### Electron microscopy.

Viruses propagated in eggs were pelleted by ultracentrifugation at 35,000 rpm for 90 min. The pellets were suspended in TNE buffer, (50 mM Tris-HCl [pH 7.4], 100 mM NaCl, 0.1 mM EDTA) layered onto a 20/40/60% sucrose step gradient, and centrifuged at 28,000 rpm for 2 h. The virus band was collected and pelleted by ultracentrifugation as described above. The obtained virions were stained using standard procedures and observed under an electron microscope at 80 kV (61).

Ultrathin-section electron microscopy was performed as described previously (62). Briefly, virus-infected cells were fixed with 2.5% glutaraldehyde in 0.1 M cacodylate buffer. The cells were then dehydrated with a series of ethanol gradients followed by propylene oxide. Thin sections were stained with 2% uranyl acetate and Reynold’s lead and observed under an electron microscope at 80 kV.

### Statistical analysis.

Quantitative data are presented as the means and standard deviations (SD) for at least three biological replicates. Data were statistically analyzed with a one-way ANOVA followed by a *t* test by using GraphPad Prism 8.0 software. Statistical parameters are reported in the figures and figure legends. *P* values of <0.05 were considered statistically significant.
