# Supplementary material for: SUMOylation of Matrix Protein M1 and Filamentous Morphology Collectively Contribute to the Replication and Virulence of Highly Pathogenic H5N1 Avian Influenza Viruses in Mammals
Source: J Virol. 2022 Feb 23;96(4):e01630-21. doi: 10.1128/jvi.01630-21 (PMC8865470; doi:10.1128/jvi.01630-21)
Supplement: Supplemental file 1 — Fig S1 to S4. Download jvi.01630-21-s0001.pdf, PDF file, 0.4 MB [file jvi.01630-21-s0001.pdf]

Supporting information

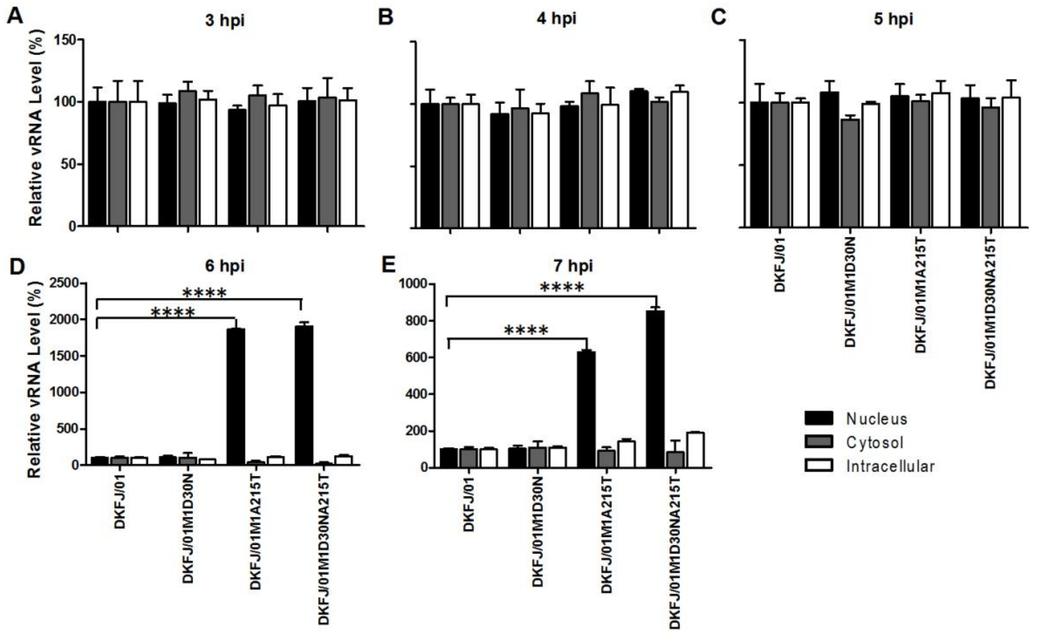

Figure S1. vRNA distribution in the nucleus, cytoplasm, and cells. MDCK cells were infected with DKFJ/01, DKFJ/01-M1D30N, DKFJ/01-M1A215T, or DKFJ/01-M1D30N/A215T virus at an MOI of 5. (A-E) The cells were collected 3, 4, 5, 6, and 7 hpi, and the nuclear and cytoplasmic components were separated. Real-time qPCR was performed for the cells. The subcellular localization of the DKFJ/01 virus in the cytoplasm, nucleus, and cells, in general, was 100% at different time points. The values shown are the means ± SD of three independent experiments. Statistical analysis was performed by using one-way ANOVA with GraphPad Prism 8 software. ****, $P < 0.0001$.

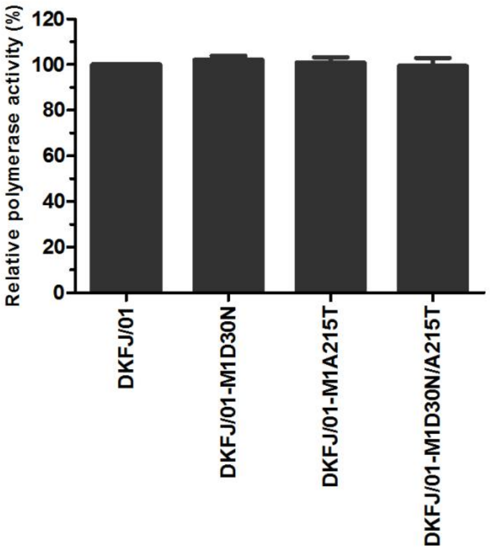

Figure S2. Effect of the mutations of M1 on activities of ribonucleoprotein (RNP) in a minigenome assay. 293T cells were cotransfected with expression plasmids for PB2, PB1, PA, NP, and wild-type or mutant M1. At 48 h posttransfection, the cells were subjected to the dual-luciferase assay. The value for the wild-type M1 was set as 100%. The data from three independent experiments are shown. Error bars represent standard deviations of three independent experiments.

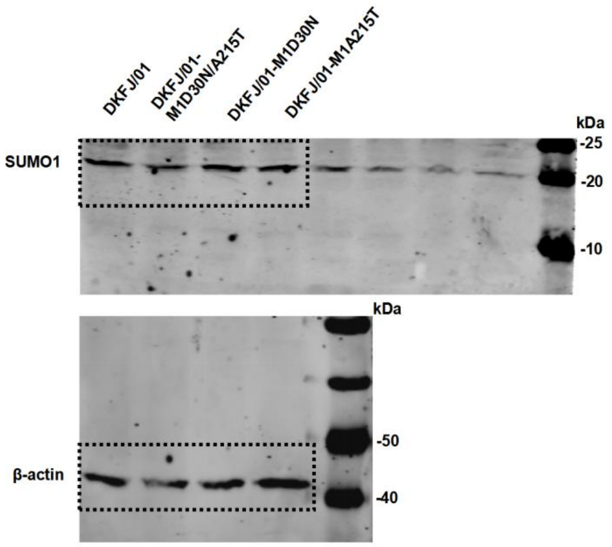

Figure S3. Detection of the SUMO1 protein in the virus-infected cells. MDCK cells were infected with the wild-type or mutant viruses at an MOI of 1, and the infected cells were collected at 10 h.p.i. SUMO1 protein was then measured by Western-blot.

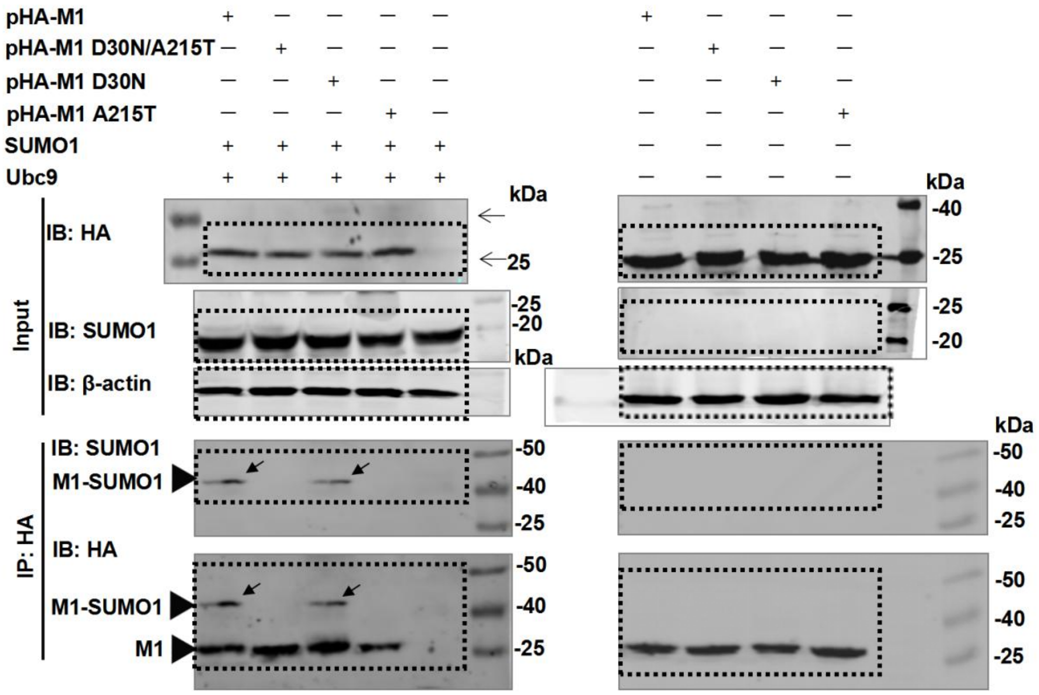

Figure S4. The mutation A215T in M1 affects the modification of the M1 protein by SUMO1. Plasmids expressing HA-tagged wild-type or mutant M1 of H5N1 influenza virus were cotransfected with Ubc9- and SUMO1-expressing plasmids into 293T cells. Forty-eight hours post-transfection, the lysates were immunoprecipitated with anti-HA agarose, followed by Western blot analysis using antibodies against HA and SUMO1.
